# Epidemiology and Genetic Diversity of *Bartonella* in Rodents in Urban Areas of Guangzhou, Southern China

**DOI:** 10.3389/fmicb.2022.942587

**Published:** 2022-07-04

**Authors:** Xin-Yan Yao, Hong Liu, Jing Sun, Yu-Qian Zhang, Zhi-Hang Lv, Xue-Lian Zhang, Jian-Wei Shao

**Affiliations:** School of Life Science and Engineering, Foshan University, Foshan, China

**Keywords:** *Bartonella*, epidemiology, genetic diversity, rodents, urban areas

## Abstract

*Bartonella* spp. are gram-negative bacteria that can infect a wide spectrum of mammals. Rodents are considered to be the natural reservoir of many *Bartonella* species that are transmitted by various blood-sucking arthropods. The close contact between rodents and humans in urban areas increased the chance of transmitting rodent-borne *Bartonella* to humans. Investigation of the epidemiological characteristics of *Bartonella* infection in rodents is of great significance for the prevention and control of human Bartonellosis. In this study, rodents were captured to monitor the prevalence of *Bartonella* in urban areas of Guangzhou city. Six official or candidate species of *Bartonella*, including two confirmed zoonotic species, were detected with an overall prevalence of 6.4% in rodents captured herein. In addition, *Rattus norvegicus* was the predominant host species for *Bartonella* infection, and *B. queenslandensis* was the dominant species circulating in rodents in these areas. These results provide insights into the prevalence and genetic diversity of *Bartonella* species circulating in rodents in the urban areas of Guangzhou, and also urged the surveillance of rodent-associated *Bartonella* species in these areas.

## Introduction

*Bartonella* spp. are gram-negative bacteria that belong to the genus of α-proteobacteria within the family *Bartonellaceae* (Anderson and Neuman, 1997). They can infect a wide range of mammals and are mainly transmitted by blood-sucking arthropods, such as fleas, lice, sandflies, biting flies, and ticks (Álvarez-Fernández et al., 2018). More than 50 validated species of *Bartonella* have been identified from domestic and wild animals, including horses, cattle, deer, sheep, rodents, and bats (Chang et al., 2000; Valentine et al., 2007; Okaro et al., 2017). As far as we know, at least 18 species of *Bartonella*, including *Bartonella alsatica, B. ancashensis, B. bacilliformis, B. clarridgeiae, B. doshiae, B. elizabethae, B. grahamii, B. henselae, B. koehlerae, B. kosoyi, B. mayotimonensis, B. quintana, B. rattimassiliensis, B. rochalimae, B. tamiae, B. tribocorum, B. vinsonii*, and *B. washoensis*, have been recognized to be associated with human diseases (Gundi et al., 2004; Kosoy et al., 2010; Kandelaki et al., 2016; Vayssier-Taussat et al., 2016; Okaro et al., 2017; Von Loewenich et al., 2019). Infection of different species of *Bartonella* can cause human diseases with different clinical manifestations, sometimes even fatal in immunocompromised patients (Mosepele et al., 2012).

Rodents represent the most abundant taxonomic order with some special ecological traits, such as wide geographic distribution and intimate interactions with humans and livestock. Thus, rodents play key roles in the transmission of a large variety of pathogenic agents of infectious diseases, including *Bartonella* (Meerburg et al., 2009). Among all known reservoir hosts of *Bartonella* species, rodents are considered the primary host, and *Bartonella* spp. in rodents have been reported in Asia (Li et al., 2015; Saengsawang et al., 2021; Liu et al., 2022), Africa (Hatyoka et al., 2019), Europe (Divari et al., 2020; Szewczyk et al., 2021), Americas (De Sousa et al., 2018; Müller et al., 2020), and Australia (Dybing et al., 2016; Egan et al., 2021). Approximately 90 species of rodents are known to be associated with more than 22 different species of *Bartonella*, ten of which have been confirmed to cause human infection (Daly et al., 1993; Welch et al., 1999; Birtles et al., 2002; Kosoy et al., 2003, 2008, 2010; Lin et al., 2008; Kandelaki et al., 2016; Vayssier-Taussat et al., 2016). Interestingly, many different rodent species have been reported to be infected with different *Bartonella* spp. in high prevalence globally (Gutiérrez et al., 2015). The high genetic diversity and infection rate of *Bartonella* in rodents emphasized the important roles that rodents played in the maintenance and transmission of *Bartonella*.

A previous study showed high infection rates, ranging from 4 to 50%, of *Bartonella* in rodents in China (Gutiérrez et al., 2015). Importantly, several studies reported the high prevalence of *Bartonella* species in rodents in the western (Rao et al., 2021), eastern (Qin et al., 2019), southern (Su et al., 2020), southeastern (Liu et al., 2022), southwest (Li et al., 2007), and northeastern China (Li et al., 2015), indicating the wide geographical distribution of *Bartonella* in rodents. However, most of the previous studies were conducted in the field environment. Information about the epidemiology of *Bartonella* spp. in urban areas where rodents have more chance of contact with humans is limited. Guangzhou, the provincial capital city of Guangdong province and the largest city in southern China, is located in the tropical and subtropical regions with a warm and moist climate (Wei et al., 2018), an ideal habitat for rodents. However, little information about the epidemiology of *Bartonella* in rodents in urban areas of Guangzhou is available. Therefore, in this study, we collected rodent samples in urban areas of Guangzhou to investigate the epidemiology of *Bartonella* spp. in rodents. The results of this study will provide insights into the genetic diversity and prevalence of *Bartonella* in Guangzhou, and also formulate the prevention and control strategies for *Bartonella* infections in this city.

## Materials and Methods

### Rodent Samples: Collection and Processing

**Figure 1:**
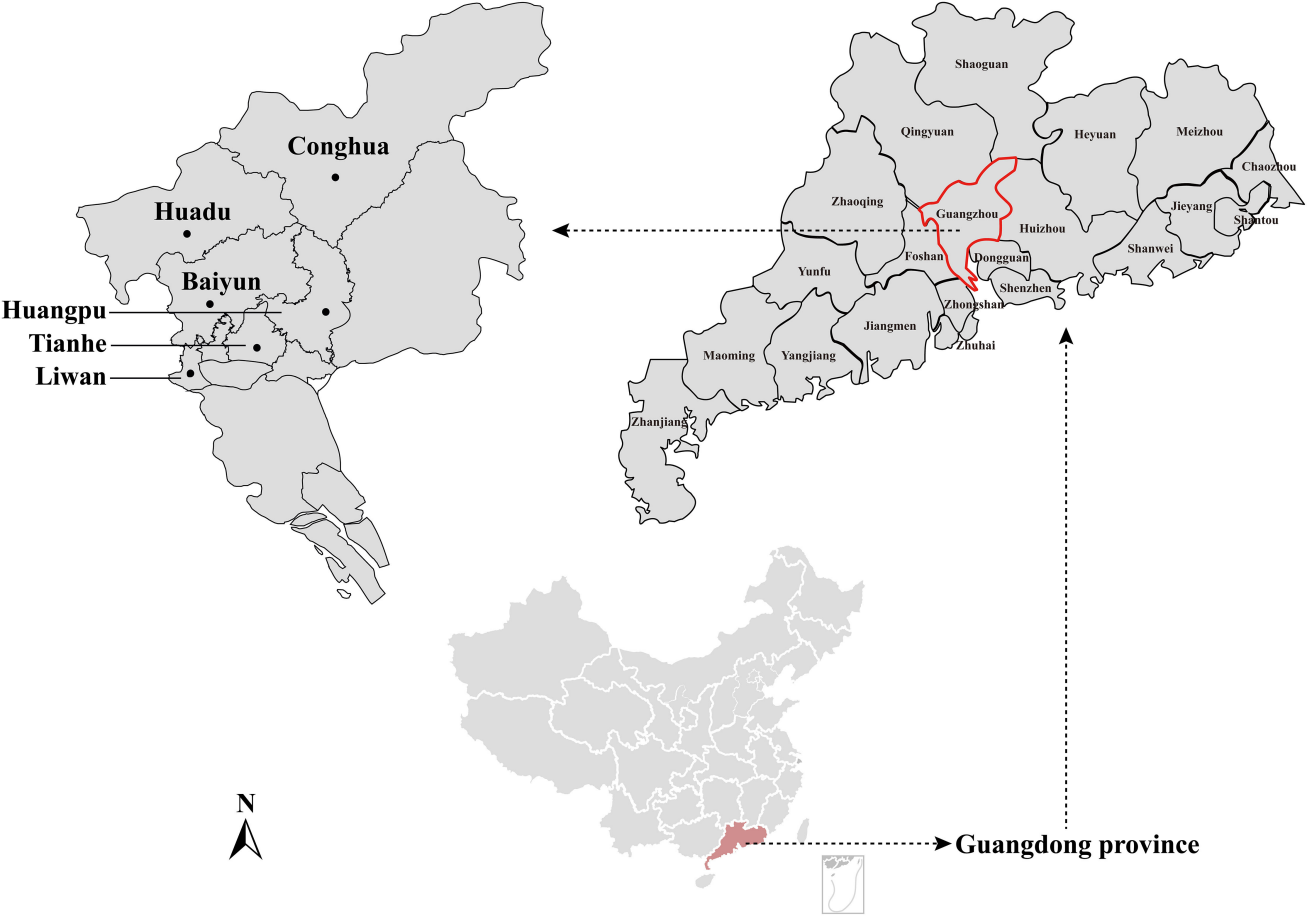
Geographic maps showing the location of sampling sites from where the rodents were captured in this study. This map was plotted by combination of Surfer software version-4 (Golden Software, USA) and Adobe illustrator version CC2017 (Adobe, USA). The black dots indicate the sampling regions in this study.

Rodents were captured using live-capture traps baited with cooked food in the urban areas of six districts (Liwan, Huadu, Tianhe, Huangpu, Baiyun, and Conghua) in Guangzhou city in 2020 (Figure 1). All rodents were euthanized, and the species, age, and sex were identified by trained field biologists, and the species were further confirmed by the sequence analysis of the mt-*cyt* b gene (Guo et al., 2013). Spleen samples were aseptically obtained immediately after euthanasia and stored at −80 °C until further use.

Approximately 20 mg of spleen samples were homogenized and further subjected to extract the total DNA using a DNA extraction kit (OMEGA, Doraville, CA, USA) as per the manufacturer's instructions. Then, all extracted DNA samples were used to detect the presence of *Bartonella*.

### Detection and Molecular Characterization of *Bartonella*

*Bartonella* spp. were detected by nested-PCR using the primers targeting the conserved region of 16S rRNA (*rrs*) gene of *Bartonella* species as previously described (Zhang et al., 2019). The targeted region, about 1,100 bp fragment, was suspected to be a *Bartonella*-positive sample and was further confirmed by sequencing. To better determine and characterize the species of detected *Bartonella* strains, the citrate synthase gene (*gltA*) and the RNA polymerase beta-subunit gene (*rpoB*) were also amplified from the positive samples. All primer sequences used in the present study are listed in Table 1.

**Table 1:** Primers used in this study.

**gene**	**Primers**	**Sequences (5^′^ → 3^′^)**	**Tm**	**Amplicon (bp)**
*rrs*	Bar1	AGAGTTTGATCMTGGCTCAGA	62 °C	1100
	Bar2	GTAGCACGTGTGTAGCCCA		
	Bar3	CACTCTTTTAGAGTGAGCGGCAA	65 °C	1000
	Bar4	CCCCCTAGAGTGCCCAACCA		
*gltA*	gltA-1F	GCTTCTTGTGAATCRAAAATYAC	48 °C	1100
	gltA-1R	GATCYTCAATCATTTCTTTCCA		
	gltA-2F	TTTAYCGYGGTTATCCTATYG	55 °C	1000
	gltA-2R	AATGCAAAAAGAACAGTAAACA		
*rpoB*	rpoB-F	ACAGTKATGCCGCAGGATTT	54 °C	747
	rpoB-R1	GAATAACAATACGGGTMGCAT		
	rpoB-F	ACAGTKATGCCGCAGGATTT	54 °C	742
	rpoB-R2	ACAATACGGGTHGCATCAAC		

The PCR products with the expected size of each primer set were purified using a gel extraction kit (TaKaRa, Dalian, China) after electrophoresis. The purified DNA was cloned into a pMD19-T vector (TaKaRa, China), and the resulting plasmid was used to transform into competent *E. coli* cells. Positive inserts were confirmed by PCR, and five positive clones were sequenced by the Sangon Biotechnology Company (Shanghai, China). To prevent contamination, the procedures including extraction of total DNA, preparation of PCR mix, adding the template DNA, and agarose gel electrophoresis were performed in separate rooms using dedicated pipets and filtered tips. Distilled water was used as the negative control in all the PCR amplifications.

### Sequence Comparison and Phylogenetic Analysis

Sequence assembly and manual editing were performed using the SeqMan program (DNASTAR, Madison, WI). The nucleotide (nt) sequence identities were calculated by the MegAlign program available within the Lasergene software package (version 7.1, DNAstar). All the sequences obtained in this study have been submitted to GenBank under the accession numbers ON413697–ON413715 for *rrs* gene and ON394008–ON394045 for *gltA* and *rpoB* genes, respectively.

The maximum-likelihood (ML) trees were constructed based on the general time-reversible (GTR) nucleotide substitution model and the optimized parameters of gamma (Γ)-distribution and proportion of invariable sites (i.e., GTR + Γ + I) with bootstrap support values calculated from 100 replicates implemented in MEGA X (Kumar et al., 2018).

### Statistical Analysis

Statistical Package for Social Sciences (SPSS) Version 21.0 software was used in the statistical analyses of this study, and Fisher exact test was used to calculate the *P*-value to determine the differences in *Bartonella* positive rates between sampling sites and animal hosts. A *P* < 0.05 was considered statistically significant.

### Ethics Statement

The authors confirmed that this study complies with the ethical policies of the journal, as specified in the journal's guidelines. All rodents were trapped by experienced field workers, and the sampling procedures and sample processing have been approved by the ethics committee of Foshan University.

## Results

### Rodent Trapping

A total of 296 rodents were captured in six districts of Guangzhou city in 2020 (Table 2). The captured rodents were identified into four species, including 250 *Rattus norvegicus*, 40 *R. losea*, 2 *R. tanezumi*, and 4 *Mus musculus*. *R. norvegicus* (250/296, 84.5%) was the most abundant species in urban environment in Guangzhou city. Among all rodents, the female and male were 123 and 173, respectively. In addition, the juvenile and adult rodents were 208 and 88, respectively.

**Table 2:** Prevalence of Bartonella in rodents collected in urban areas of Guangzhou.

**Parameters**	**Location**						**Sub-total (%)**	**95% CI (%)**	**χ^2^-value**	***P*** **-value**
	**Liwan**	**Huadu**	**Tianhe**	**Huangpu**	**Baiyun**	**Conghua**				
**Species**									3.736	0.291
*R.norvegicus*	0/46	11/50	5/50	–/48	2/46	1/10	19/250 (7.6)	4.3–10.9		
*R. losea*	–	–	–	–	–	40	0/40	0		
*R. tanezumi*	0/2	–	–	–	–	–	0/2	0		
*Mus musculus*	0/2	–	–	0/2	–	–	0/4	0		
**Gender**									0.003	0.571
Female	0/22	6/24	2/19	0/19	0/18	0/21	8/123 (6.5)	2.1–10.9		
Male	0/28	5/26	3/31	0/31	2/28	1/29	11/173 (6.4)	2.8–10.0		
**Age**										
Juvenile	0/44	6/21	5/38	0/29	1/40	1/36	13/208 (6.3)	3.0–9.5	0.033	0.518
Adult	0/6	5/29	0/12	0/21	1/6	0/14	6/88 (6.8)	1.5–12.1		

*Bartonella spp. DNA positive specimens/total specimens; “–” indicates that no animals were captured*.

### Prevalence of *Bartonella* in Rodents

The total DNAs extracted from the rodents' spleen were subjected to PCR targeting the *rrs* gene of *Bartonella* spp., and PCR products with expected sizes were detected in 19 samples (Table 2). Sequencing and blast analysis of the PCR products confirmed that these 19 samples were *Bartonella* positive. Overall, the prevalence of *Bartonella* spp. in this study was 6.4% (19/296, 95% CI: 3.6–9.2%). Specifically, all *Bartonella*-positive samples were exclusively detected from *R. norvegicus* (7.6%, 95% CI: 4.3–10.9%). In addition, the infection rates of *Bartonella* in female and male rodents were 6.5% (95% CI: 2.1–10.9%) and 6.4% (95% CI: 2.8–10.0%), respectively. Moreover, 6.3% (95% CI: 3.0–9.5%) of the juvenile rodents and 6.8% (95% CI: 1.5–12.1%) of adult rodents were determined as *Bartonella*-positive. Notably, no significant difference was observed associated either with the rodent species or with the gender or age of the rodents.

### Molecular Characterization of *Bartonella*

The *rrs, gltA*, and *rpoB* genes were recovered from positive samples to better characterize the species of detected *Bartonella* strains. Sequencing and blast analyses based on the *rrs* gene sequences (1,100 bp) revealed that *Bartonella* strains detected in this study were *B. queenslandensis* (*n* = 7), *B. mastomydis* (*n* = 5), *B. tribocorum* (*n* = 2), *B. rattimassiliensis* (*n* = 1), *Bartonella* sp. AA86HXZ (*n* = 3), and *Bartonella* sp. Fuji 12-1 (*n* = 1).

**Figure 2:**
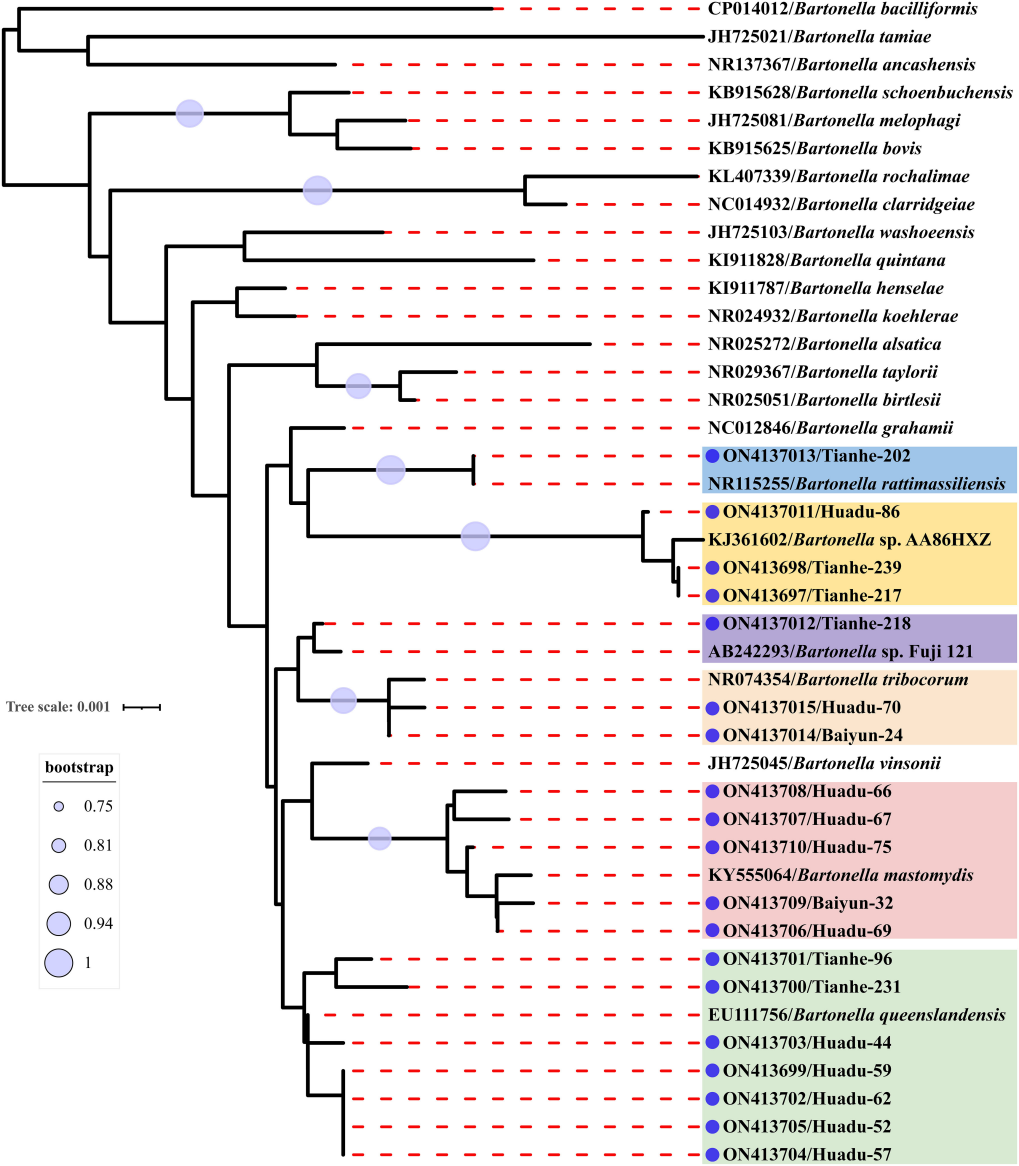
Maximum-likelihood phylogenetic tree reconstructed based on the nucleotide sequences of *rrs* gene of *Bartonella*. Bootstrap values were calculated with 100 replicates of the alignment, and only bootstrap values >70% are shown at appropriate nodes. Sequences of *Bartonella* determined in this study are marked with blue dot.

**Figure 3:**
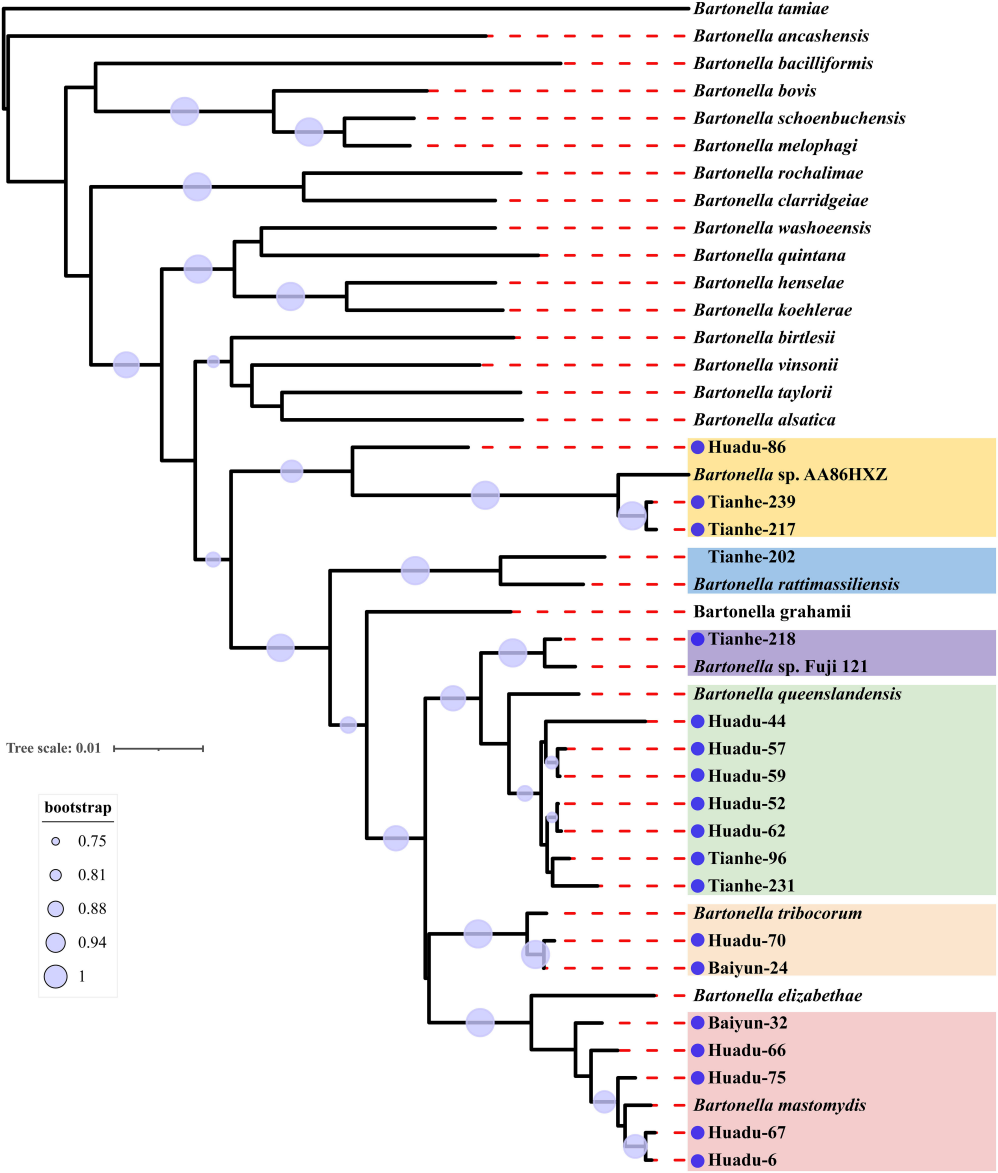
Maximum-likelihood phylogenetic tree reconstructed based on the concatenated nucleotide sequences of *rrs, gltA*, and *rpoB* genes of *Bartonella*. Bootstrap values were calculated with 100 replicates of the alignment, and only bootstrap values >70% are shown at appropriate nodes. Sequences of *Bartonella* determined in this study are marked with blue dot.

Sequence comparison analysis showed that all *rrs* gene sequences generated herein shared 98.2–100% nucleotide sequences identity with each other. The seven *rrs* gene sequences of *B. queenslandensis* obtained in the present study shared the highest sequence similarity with *B. queenslandensis* strain AUST/NH8 (EU111756), with 99.3–100% nucleotide sequences identity. Similarly, the *rrs* gene sequences of *B. mastomydis, B. tribocorum, B. rattimassiliensis, Bartonella* sp. AA86HXZ, and *Bartonella* sp. Fuji 12-1 obtained in the present study exhibited 99.5–99.9%, 99.8–100%, 100%, 99.3–99.6%, and 99.7% nucleotide sequences identical with the sequences of the corresponding species of *Batonella* under the GenBank accession number KY555064, NR074354, NR115255, KJ361602, and AB242293, respectively. Phylogenetic trees reconstructed based on the *rrs* gene sequences and the concatenated sequence of *rrs, gltA*, and *rpoB* genes showed a similar topology that these sequences determined in this study clustered together with the corresponding reference sequences, respectively (Figures 2, 3).

## Discussion

A wide range of emerging and re-emerging infectious diseases have become major global threats to human health (Dong and Soong, 2021). *Bartonella* spp. are emerging vector-borne pathogens distributed worldwide and cause global public health concerns (Mogollon-Pasapera et al., 2009). Rodents are considered the major reservoirs that harbor the largest number and the greatest diversity of *Bartonella* species (Gutiérrez et al., 2015), and the presence of many pathogenic *Bartonella* species have been confirmed (Okaro et al., 2017). Therefore, investigation of the epidemiological characteristics of *Bartonella* infection in rodents is of great significance for the prevention and control of human Bartonellosis.

In the present study, rodents were captured in urban areas of Guangzhou city, and *Bartonella* was detected with an overall prevalence of 6.4%, which was lower than the results reported previously in the urban environments in Thailand (Saengsawang et al., 2021), Argentina (De Salvo et al., 2020), and Malaysia (Blasdell et al., 2019). In addition, the prevalence of *Bartonella* in our study was lower than those in other areas in China (Li et al., 2015; An et al., 2020; Rao et al., 2021; Liu et al., 2022). The observed differences in the prevalence of *Bartonella* in rodents may be related to rodent species, habitats, and arthropod vector populations (Meheretu et al., 2013). Herein, all *Bartonella*-positive were detected from *R. norvegicus*, which is the predominant species of rodents captured in our study and also a synanthropic rodent species mostly found in the urban environments (Gardner-Santana et al., 2009; Kosoy and Bai, 2019). Particularly, fleas have been shown to play an extremely important role in the transmission and acquisition of *Bartonella* species in rodents (Silaghi et al., 2016). Rodents inhabiting urban environments have less chance of flea-parasitic infestation, thus reducing the possibility of *Bartonella* infection. However, *R. norvegicus* closely cohabitates with humans living inside buildings and enhances the likelihood of human contact with rodents, suggesting that *R. norvegicus* may be an important source of zoonotic pathogens (Firth et al., 2014). *Bartonella* species detected in this study also suggest that much more attention should be focused on the increased risk of *Bartonella* transmission to humans in urban environments.

revious data indicated that the majority of known *Bartonella* spp. are carried by rodents, some of which have been implicated as the causative agents of human diseases (Gutiérrez et al., 2015; Okaro et al., 2017). In this study, six official or candidate species of *Bartonella*, including *B. queenslandensis, B. mastomydis, B. tribocorum, B. rattimassiliensis, Bartonella* sp. AA86HXZ, and *Bartonella* sp. Fuji 12-1 were detected in rodents, indicating the genetic diversity of *Bartonella* circulating in the rodent population in the urban areas of Guangzhou city. Notably, two confirmed human-pathogenic *Bartonella* species, i.e., *B. tribocorum* and *B. rattimassiliensis*, were detected in this study, indicating that infection risks exist in human populations in urban areas that originated from rodent-associated *Bartonella*, and also highlighting the importance of the surveillance of *Bartonella* infection in rodents.

In conclusion, genetically diversified *Bartonella*, including two confirmed zoonotic species, were detected in rodents in the urban areas of Guangzhou city with an overall prevalence of 6.4%. Moreover, *R. norvegicus* was the predominant host species for *Bartonella* infection, and *B. queenslandensis* was the dominant species circulating in rodents in these areas. These results provide a better understanding of the prevalence and genetic diversity of *Bartonella* species circulating in rodents in the urban areas of Guangzhou and also urged the necessity of the surveillance of rodent-associated *Bartonella* species in these areas.

## Data Availability Statement

The datasets presented in this study can be found in online repositories. The names of the repository/repositories and accession number(s) can be found in the article/Supplementary Material.

## Ethics Statement

The animal study was reviewed and approved by Ethics Committee of Foshan University.

## Author Contributions

J-WS conceived and designed the experiments and writing—review and editing. X-YY, HL, and JS performed the experiments and analyzed the data. Y-QZ, Z-HL, and X-LZ help to collect the samples. X-YY writing—original draft preparation. All authors contributed to the article and approved the submitted version.

## Funding

This work was funded by the Key Project of Agricultural and Social Development Science and Technology Projects of Guangzhou (No. 202103000008). The funder had no role in study design, data collection and interpretation, or the decision to submit the work for publication.

## Conflict of Interest

The authors declare that the research was conducted in the absence of any commercial or financial relationships that could be construed as a potential conflict of interest.

## Publisher's Note

All claims expressed in this article are solely those of the authors and do not necessarily represent those of their affiliated organizations, or those of the publisher, the editors and the reviewers. Any product that may be evaluated in this article, or claim that may be made by its manufacturer, is not guaranteed or endorsed by the publisher.
