# Supplementary figures and images for: Epidemiology and Genetic Diversity of Bartonella in Rodents in Urban Areas of Guangzhou, Southern China
Source: Front Microbiol. 2022 Jul 4;13:942587. doi: 10.3389/fmicb.2022.942587 (PMC9289675; doi:10.3389/fmicb.2022.942587)

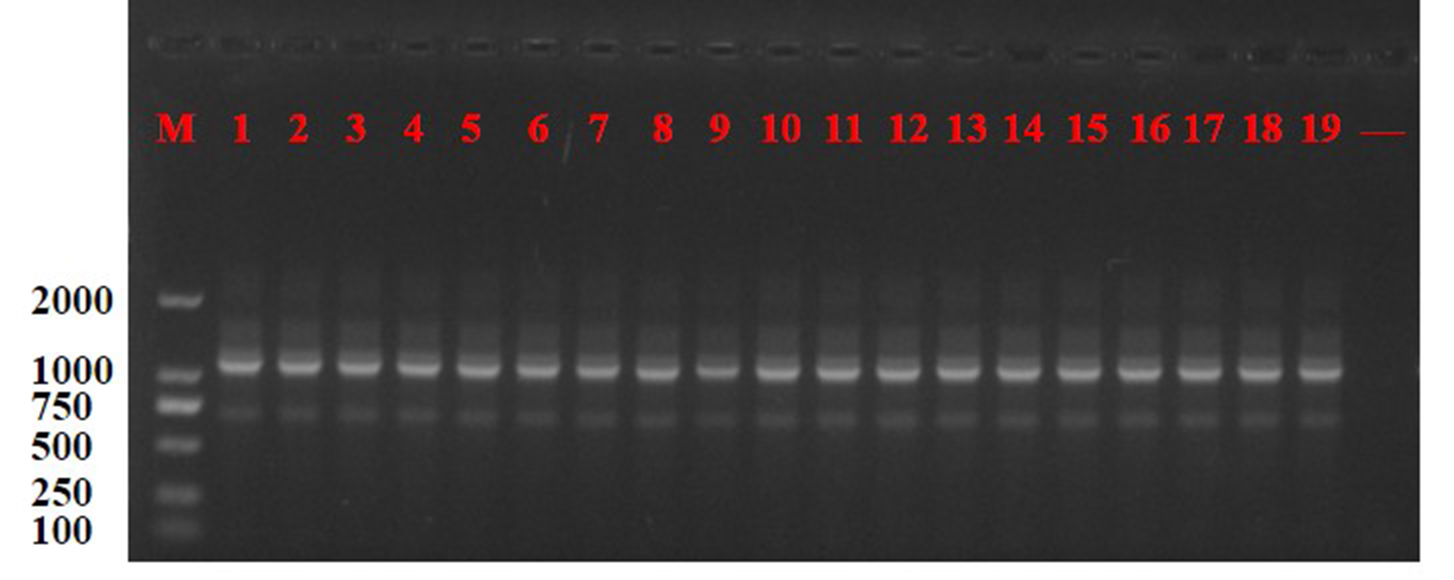

Supplement: Supplementary file 2 [file Image_1.JPG]
